# The Lifetime of Sequential Memory Traces in the Absence of Language

**DOI:** 10.1111/cogs.70095

**Published:** 2025-08-05

**Authors:** Laura Ordonez Magro, Leonardo Pinto Arata, Joël Fagot, Jonathan Grainger, Arnaud Rey

**Affiliations:** ^1^ Psychological Sciences Research Institute Université catholique de Louvain; ^2^ Aix‐Marseille Université CNRS, CRPN; ^3^ Aix‐Marseille Université CNRS, LIS; ^4^ Aix‐Marseille Université CNRS, ILCB; ^5^ Station de Primatologie‐Celphedia CNRS

**Keywords:** Statistical learning, Associative learning, Sequence learning, Memory trace

## Abstract

Statistical learning allows us to implicitly create memory traces of recurring sequential patterns appearing in our environment. Here, we study the dynamics of how these sequential memory traces develop in a species of nonhuman primates (i.e., Guinea baboons, *Papio papio*) that, unlike humans, cannot use language and verbal recoding strategies to strengthen these memory traces. We test a group of Guinea baboons in a Hebb visuo‐motor pointing task in which a target sequence is repeated with random sequences inserted between repetitions. In this study, we systematically manipulate the interval between two repetitions of the target sequence by varying the number of interposed random sequences. We found that baboons can learn repeated visuo‐motor sequences, even when the repetitions are separated by six random sequences. Our results also suggest that the learning curve of the target sequence best fits a logarithmic function. The present study, therefore, provides a quantitative assessment of the development of a sequential memory trace as a function of repetition spacing and without the use of verbal recoding strategies.

## 1 Introduction

Statistical learning (SL), the ability to encode recurrent sequential patterns appearing in our environment, is assumed to be a critical building block for the development of a wide range of cognitive abilities in humans, such as sequence learning, motor planning, spoken and written language acquisition, and social cognition (Frost, Armstrong, & Christiansen, 2019). Since this phenomenon is involved in the perception and learning of many forms of knowledge, understanding its underlying mechanisms is of utmost importance for advancing general theories of learning in cognitive science (Bogaerts, Frost, & Christiansen, 2020).

Over the past two decades, in parallel with the growing number of studies aimed at understanding the mechanisms supporting regularity extraction in the environment (see Christiansen, 2019, for a review), several computational models have been developed to account for these processes (e.g., Boucher & Dienes, 2003; Cabiddu, Bott, Jones, & Gambi, 2023; Elman, 1990; Frank, Goldwater, Griffiths, & Tenenbaum, 2010; French, Addyman, & Mareschal, 2011; Jessop, Pine, & Gobet, 2025; McCauley & Christiansen, 2019; Monaghan & Christiansen, 2010; Perruchet & Vinter, 1998; Robinet, Lemaire, & Gordon, 2011; Servan‐Schreiber & Anderson, 1990). For instance, the Simple Recurrent Network (SRN; Elman, 1990), a connectionist neural network trained to predict a given item of a sequence based on previous items, suggests that a sequence is learned through the adjustment of weights in the network. As a result, items that are frequently encountered together develop stronger connections with each other. PARSER (Perruchet & Vinter, 1998), another influential model of SL, assumes that such frequently associated components are grouped together and temporarily encoded as chunks in memory. Importantly, each time a chunk is reencountered, its memory trace is strengthened, and conversely, it progressively decays if it is not encountered again, until it entirely vanishes.

While there is a growing body of evidence that sheds light on the mechanisms that are involved in SL, evidence regarding the temporal dynamics of memory trace formation and survival in SL is scarce. Indeed, little is known about when exactly a sequential memory trace that is not repeatedly processed can no longer benefit from repetition. The SRN model predicts that, once a target sequence is processed, the resulting changes in connection weights are stored in the network and will not decay. However, this trace in memory may eventually suffer interference from other traces created between two repetitions of the target sequence, thus diminishing the strength of the trace, without erasing it completely. Conversely, PARSER predicts that if the delay between two repetitions of a target sequence is too great, its trace in memory will decay and eventually disappear.

In everyday life, most sequential information that is learned is not necessarily frequently encountered in the environment. For instance, infants are able to learn multiple words very quickly even when they are not constantly exposed to them (Pinker, 1994; Swingley, 2008). Based on this idea, Page, Cumming, Norris, McNeil, and Hitch (2013) conducted one of the rare studies that investigates the effect of repetition spacing (i.e., the role of the number of random sequences that intervene between two repetitions of a target sequence) in the learning of sequential material. They used a variant of Hebb's repetition paradigm (Hebb, 1961) in which adult humans are presented with sequences of digits to be recalled immediately. In Hebb's seminal study, a target sequence (hereafter, the Hebb sequence) is repeated every third trial and interposed by random (filler) sequences. In their study, Page et al. presented their participants with sequences of syllables in which repetitions were spaced at every 6th, 9th, and 12th trial. They observed that the learning rate for the repeated sequence was substantial and essentially equivalent for all three spacing conditions. These findings suggest that learning is still possible when repetitions are widely spaced and that memory traces that are not constantly refreshed do not vanish rapidly.

Note, however, that unlike in other species, SL in humans can be based on the ability to verbally recode nonverbal material (Baddeley, 2003) and to internally repeat sequences, for instance, by using the phonological loop (Aboitiz, Aboitiz, & García, 2010). These verbal consolidation strategies are known to play a key role in maintaining and refreshing information in memory and thus have an undeniable impact on memory performance (Barrouillet & Camos, 2014). Given the close link between memory and language, it is difficult to disentangle memory‐specific from language‐related mechanisms. One possible way to avoid the presence of any language bias when examining fundamental SL mechanisms is to test species that lack language. Indeed, the ability to learn repeated and frequent co‐occurrences has been observed not only in humans, but also in nonhuman primates such as tamarins (Hauser, Newport, & Aslin, 2001), macaques (Wilson, Smith, & Petkov, 2015), and baboons (Fagot et al., 2019; Malassis, Rey, & Fagot, 2018; Minier, Fagot, & Rey, 2016; Rey, Perruchet, & Fagot, 2012), indicating that regularity extraction can occur in the absence of language and verbal recoding strategies. These studies in nonhuman primates, therefore, suggest that SL is probably based on domain‐general associative learning mechanisms (Rey, Minier, Malassis, Bogaerts, & Fagot, 2019), such as chunking (Perruchet, Tyler, Galland, & Peereman, 2004; Pinto Arata, Tosatto, & Rey, 2024; Tosatto, Fagot, Nemeth, & Rey, 2022).

Chunking refers to the cognitive process of grouping frequently occurring items into larger processing units, known as chunks (Gobet, Lloyd‐Kelly, & Lane, 2016), to overcome memory limitations (Mathy & Feldman, 2012; Miller, 1956), and to facilitate learning across domains (Gobet et al., 2001). This mechanism is considered central to sequence learning, both in the context of the Hebb repetition effect (Musfeld, Dutli, Oberauer, & Bartsch, 2024; Page & Norris, 2009) and SL (Christiansen, 2019; Isbilen, McCauley, Kidd, & Christiansen, 2020). Notably, chunking has been proposed to rely on Hebbian learning principles (Perruchet & Vinter, 2002), and both mechanisms have been incorporated into recent computational models of SL (e.g., Fonollosa, Neftci, & Rabinovich, 2015; Tovar & Westermann, 2023), suggesting that they may represent two complementary aspects of the same learning process (Rey, 2024).

In the present study, we sought to investigate the effect of repetition spacing on the formation of sequential memory traces in the absence of verbal recoding strategies. To do so, we tested baboons using an adaptation of the Serial Reaction Time task (SRT, Nissen & Bullemer, 1987) combined with the advantage of the Hebb repetition paradigm, which allows to control for the spacing between repetitions by inserting a varying number of random filler sequences between each repetition of the target sequence. In this task, baboons had to touch a red circle that moved on a touchscreen in a fixed sequence of three target locations (selected from a 3×3 matrix of nine locations). Between two repetitions of the repeated sequence, we inserted either one filler sequence (1F‐Condition), three filler sequences (3F‐Condition), six filler sequences (6F‐Condition), or no filler sequence (0F‐Condition).

We decided to use an SRT task because this type of task has been successfully used to study sequence learning in both human (Malassis et al., 2018) and nonhuman primates (Minier et al., 2016; Rey et al., 2022; Yeaton, Tosatto, Fagot, Grainger, & Rey, 2023). In addition, the SRT task has been effectively combined with the Hebb repetition paradigm to study the effects of interference on memory forgetting (Ordonez Magro, Fagot, Grainger, & Rey, 2022). Recent comparative evidence suggests that both populations exhibit similar chunking dynamics during learning, with humans showing faster learning rates, likely due to verbal recoding abilities that allow for the explicit encoding of presented regularities (Rey et al., 2019; Tosatto et al., 2023).

In light of these findings, we hypothesized that baboons would develop long‐term memory traces for repeated sequences. If baboons can extract the sequence structure, we should observe a decrease in response times (RTs) for the second and third elements, as these can be predicted from the first element. However, unlike humans, whose performance appears to be largely unaffected by repetition spacing (Page et al., 2013), we expected baboon performance to decline with increasing spacing, as the memory trace would be reinforced less frequently in the absence of verbal recoding abilities. Specifically, the decrease in RTs for the second and third elements should be smaller with greater spacing. This study was designed to provide a quantitative assessment of the time course of both the formation and survival of a sequential memory trace in the absence of verbal recoding strategies. More importantly, by studying the effect of the spacing between two repetitions on the strength of the memory trace (measured by the evolution of RTs on predictable positions), we should be able to see whether, as PARSER predicts, this trace tends to disappear for spacings that are too large, or whether, as SRN predicts, something of the trace remains so that with each repetition, the sequential memory trace grows stronger.

## 2 Method

### 2.1 Participants

We tested 25 Guinea baboons (*Papio papio*, 16 females, age range 2.33–24.41 years) living in a social group at the CNRS primate facility in Rousset (France). The baboons were housed in a 700 m^2^ outdoor enclosure with access to indoor housing. Water was provided ad libitum during the test, and the monkeys received their normal food ration of fruits every day at 5 p.m.

### 2.2 Apparatus

Baboons had free access to 14 Automated Learning Devices for Monkeys (ALDM, Fagot & Bonté, 2010; Fagot & Paleressompoulle, 2009) equipped with touch screens and a food dispenser. When entering the ALDM test box, baboons were identified by microchips implanted in each arm. The system saved the last trial the baboon had achieved before leaving the box, allowing them to continue the task later where it had stopped. The experiment was controlled by E‐Prime 2.0 software (Psychology Software Tools, Pittsburgh, PA). All the baboons were familiar with touch screen experimentation.

### 2.3 Materials and procedure

**Fig. 1:**
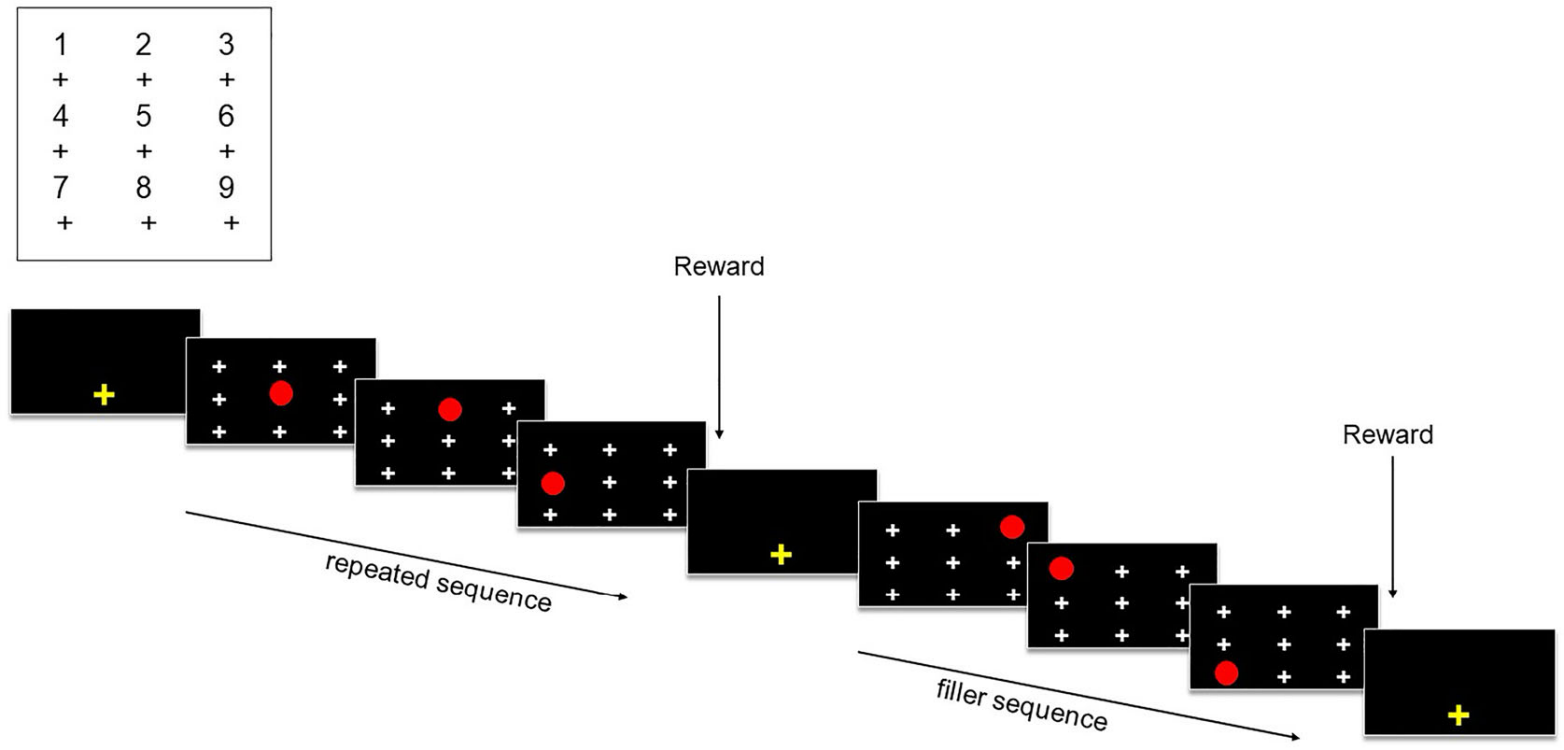
Representation of the touch screen with the nine locations (above) and example of the experimental display and stimuli presentation for repeated and random (filler) sequences (below).

To begin a trial, baboons had to press the yellow fixation cross centered at the bottom of the screen. After pressing the cross, they saw a black screen that was divided into an invisible matrix of (3×3) cells, each containing a white cross in its center (see Fig. 1).

In the task, baboons must touch sequentially one red circle that moved on the screen in sequences of three target locations. When the baboon touched the first target position, it disappeared and was replaced by the white cross. The red circle then appeared at the second position and had again to be touched before being presented with the last position of the sequence, where a last touch was required. A reward (grains of dry wheat) was delivered at the end of each sequence of three correct touches. In case of an error (i.e., the participant touched another location than the target one or failed to touch the screen within a time period of 5000 ms), a green screen was displayed for 3000 ms as a marker of failure (see the OSF repository for a video of the procedure).

The task began with a familiarization phase during which baboons were presented with random sequences of three positions. The test phase began when the baboon achieved a performance higher than 80% correct within three consecutive blocks of 100 trials. RTs between the appearance of the red circle and the participant's touch of each of the three positions for each sequence were recorded and served as the main dependent variable. The task was composed of four conditions, which differed in the number of filler random sequences introduced between the repeated target sequence. Participants first carried out the 1F‐Condition, followed by the 3F‐Condition, then the 6F‐Condition, and the 0F‐Condition. To be presented with a total of 1000 repetitions of the repeated sequence in each condition, baboons performed 10 blocks of 200 trials in the 1F‐Condition, 10 blocks of 400 trials in the 3F‐Condition, 10 blocks of 700 trials in the 6F‐Condition, and 10 blocks of 100 trials in the 0F‐Condition. To avoid learning effects across conditions, baboons were presented with a different repeated sequence in each condition (see Table 1 for an example). Note that repeated and filler sequences were nonoverlapping sequences, because the two sequence types used different locations (i.e., when the repeated sequence was composed of the locations 5‐2‐4, these locations were never used in the filler sequences; see also Ordonez Magro et al., 2022).

**Table 1:** Example of sequences presented in the four conditions for a given participant

Participant	1F‐Condition	3F‐Condition	6F‐Condition	0F‐Condition
Subject 1	7‐8‐1	3‐9‐6	4‐9‐2	**2‐7‐6**
	**6‐9‐2**	9‐6‐7	1‐8‐5	**2‐7‐6**
	4‐5‐1	6‐9‐8	9‐1‐8	**2‐7‐6**
	**6‐9‐2**	**5‐2‐4**	5‐2‐8	**2‐7‐6**
	…	3‐1‐7	5‐8‐9	…
		3‐8‐6	8‐2‐4	
		7‐9‐1	**7‐6‐3**	
		**5‐2‐4**	9‐2‐1	
		…	2‐5‐1	
			5‐1‐8	
			8‐4‐9	
			4‐5‐2	
			1‐4‐8	
			**7‐6‐3**	
			…	

*Note*. Digits correspond to the nine screen locations. Repeated sequences are bolded.

In this paradigm, learning is measured by comparing the RTs between the three positions of the sequence. In a repeating three‐position sequence such as 5‐2‐4, location 5 is always followed by location 2, and location 2 is always followed by location 4. Thus, location 5 (Position 1) should remain unpredictable over trials due to the previously presented random sequences. Location 2 (Position 2) should benefit from the systematic presence of the 5 presented just before, while location 4 (Position 3) should further benefit from the cumulative contextual information provided by the two preceding positions (Elman, 1990; Minier et al., 2016). If learning occurs, we should observe a *predictability* effect (Pinto Arata et al., 2024; Rey et al., 2020), reflected in faster RTs for Position 2 over Position 1 and even faster RTs for Position 3 compared to 2 (Pinto Arata et al., 2024; Rey et al., 2020).

### 2.4 Design of the sequences

To control for the motor difficulty of the transitions between positions of the triplet sequence to be produced, we used data from a prior study conducted on 13 baboons. In this study, baboons had to produce 1000 trials, each composed of a random sequence of six positions. Based on these random trials, a baseline measure was computed for all possible transitions from one location to another by calculating the mean RTs for each transition (e.g., from position 1 to 9) over all 13 baboons, resulting in a 9×9 matrix of mean RTs (see Appendix A). This baseline measure corresponds to the mean time it takes the baboons to move their hand from one location to another, when the transition is unpredictable (i.e., random). It thus provides a good estimate of the motor difficulty of moving from one position to another.

Importantly, to accurately assess learning, the mean RTs of the first and the second transition of the repeated sequences must be similar before any exposition to the repeated sequence (i.e., the first Transition Time, TT1, from Position 1 to Position 2, and the second Transition Time, TT2, from Position 2 to 3). Therefore, using these baseline measures, we computed TT1 and TT2 for all possible 504 triplets, and we selected a set of four triplets (serving as repeated sequences) with the smallest difference between TT1 and TT2 (see Appendix B). For each monkey, each of the four triplets was used in random order in each of the four spacing conditions.

## 3 Results

To ensure that all the spacing conditions were comparable, the analysis focused only on the baboons that completed all the conditions. Based on this criterion, we retained the 12 baboons (7 females, age range 4.33–22.66 years) that completed all four conditions. Baboons obtained a mean accuracy level of 97.34% in the 1F‐Condition, of 97.86% in the 3F‐Condition, of 97.62% in the 6F‐Condition, and of 96.76% in the 0F‐Condition. Incorrect trials were removed from the dataset. RTs for each of the three possible positions and for the 1000 trials were divided into 10 blocks of 100 trials. We normalized the data by removing RTs greater than 800 ms and then performed a recursive trimming procedure to exclude RTs greater than 2 standard deviations from the mean for each of the three possible positions in a block for each baboon (13.34%, 14.06%, 13.79%, and 13.24% of the data for conditions 1F, 3F, 6F, and 0F).^1^

To measure learning of the repeated sequence, we looked at the evolution of RTs at each of the three positions in the sequence. To adjust for the differences that may appear between positions at the very start of the experiment, we systematically calculated the difference between mean RTs obtained at each block and that obtained at the first block, for every monkey and every position. Data analysis was performed with the R software (version 4.4.1) using linear mixed‐effects models (LMEs) fitted with the lme4 package (version 1.1‐35.5; Bates, Mächler, Bolker, & Walker, 2015). Separate models were fitted for each spacing condition. Each model included Position (1 to 3), Block (1 to 10), and their two‐way interaction as fixed effects, and participants as random effects. We used the maximal random‐effect structure that allowed convergence. Position was coded using Helmert contrasts (i.e., P1: 0.7 0.0; P2: −0.3 0.5; P3: −0.3 −0.5) to compare Position 1 with both Positions 2 and 3, simultaneously, and Position 2 with Position 3. Block was mean‐centered to reduce collinearity. RTs were log‐transformed prior to each analysis to reduce skewing. Fixed effects were deemed reliable if |*t*| > 1.96 (Baayen, 2008). The results of the LMEs are summarized in Table 2.

**Table 2:** Summary of each mixed model for all spacing conditions

	0F			1F		
Predictors	β	95% CI	*t*	β	95% CI	*t*
Intercept	−53.34	[−61.46, −45.21]	−**12.91**	−35.94	[−46.07, −25.82]	−**6.98**
P1 versus P2‐P3	35.60	[21.98, 49.22]	**5.14**	35.20	[24.98, 45.42]	**6.77**
P2 versus P3	38.83	[10.63, 67.03]	**2.71**	33.22	[15.76, 50.68]	**3.74**
Block	−8.14	[−9.04, −7.23]	−**17.70**	−7.11	[−8.65, −5.58]	−**9.11**
P1 versus P2‐P3: Block	6.52	[4.60, 8.43]	**6.68**	5.63	[4.11, 7.15]	**7.27**
P2 versus P3: Block	4.86	[2.65, 7.08]	**4.32**	2.42	[0.66, 4.18]	**2.70**

	3F			6F		
Predictors	β	95% CI	*t*	β	95% CI	*t*
Intercept	−28.41	[−36.88, −19.93]	−**6.59**	−19.91	[−27.82, −12.01]	−**4.96**
P1 versus P2‐P3	20.25	[10.72, 29.77]	**4.18**	20.46	[5.01, 35.91]	**2.61**
P2 versus P3	17.88	[1.14, 34.63]	**2.10**	9.77	[−6.23, 25.78]	1.20
Block	−6.14	[−7.45, −4.84]	−**9.26**	−4.30	[−6.16, −2.45]	−**4.56**
P1 versus P2‐P3: Block	5.47	[4.19, 6.74]	**8.44**	4.29	[3.14, 5.43]	**7.35**
P2 versus P3: Block	3.66	[2.19, 5.13]	**4.89**	2.53	[1.21, 3.86]	**3.76**

*Note*. Effects are considered significant when *t* > |1.96| and are highlighted in bold.

Abbreviation: P, position.

**Fig. 2:**
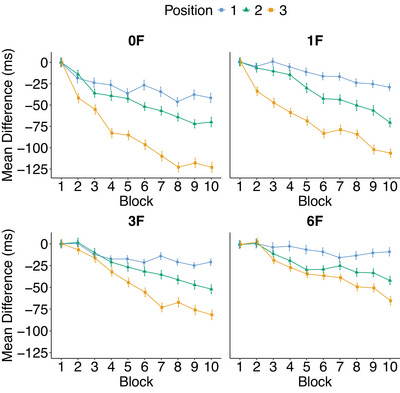
Mean reaction times differences over all baboons between each Block (from Block 1 to 10) and Block 1 for all positions in the sequence (from Position 1 to 3) in all four spacing conditions (0F, 1F, 3F, and 6F). Error bars indicate 95% confidence intervals.

The evolution of the difference between mean RTs for all Blocks and mean RTs for Block 1 for all spacing conditions (i.e., 0F, 1F, 3F, and 6F) and all positions in the repeated sequence (i.e., from Position 1 to 3) is presented in Fig. 2. Our results showed a significant effect of Block, with RTs decreasing across blocks in all spacing conditions. Consistent with previous findings, we found that baboons produced faster RTs for predictable positions (i.e., Positions 2 and 3) compared to unpredictable positions (i.e., Position 1) in all spacing conditions. Moreover, the difference in RTs between predictable positions and unpredictable positions increased throughout the blocks, as indicated by the significant positive interaction between Position 1 and the average of Positions 2 and 3, and Block. Our results also showed a processing advantage for items in Position 3 over those in Position 2 and a significant increase of this difference across blocks for all spacing conditions.

To examine the influence of repetition spacing on sequence learning, we computed a predictability score for each spacing condition by calculating the difference between the RTs for the unpredictable position (i.e., Position 1) and the mean RT for the predictable positions of the triplet for each block (i.e., Positions 2 and 3). Here, a positive score reflects learning of the predictable positions and, therefore, learning of the sequence (Pinto Arata, Ordonez Magro, Ramisch, Grainger, & Rey, 2024). We performed an LME on these predictability scores, using spacing condition and block as fixed effects, along with the interaction between these variables, and by‐subject random intercepts, as well as by‐subject random slopes for block. The spacing condition was coded using repeated contrast coding to perform pairwise comparisons. We found higher predictability scores in the 0F‐Condition compared to the 1F‐Condition, *b* = −0.04, 95% CI [−0.06, −0.02], *t* = −4.44, and higher scores in the 1F‐Condition compared to the 3F‐Condition, *b* = −0.05, 95% CI [−0.07, −0.03], *t* = −4.91. There was no difference between the 3F‐ and 6F‐Conditions. We also observed a significant effect of Block, *b* = 0.02, 95% CI [0.01, 0.02], *t* = 8.24, indicating an increase in predictability scores across blocks.

**Fig. 3:**
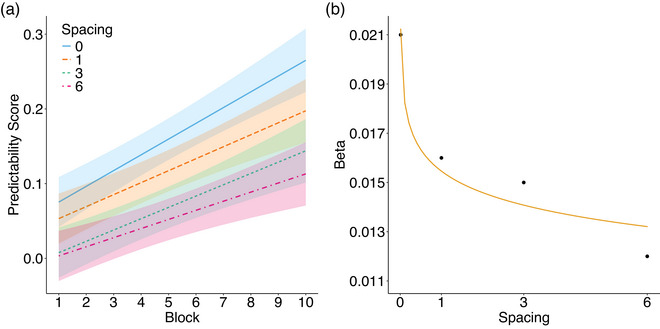
Panel a: Comparison of predictability scores across blocks for repeated sequences in all the spacing conditions. Continuous lines represent the best linear fit, and color‐shaded areas indicate 95% confidence intervals around linear regression lines. Panel b: Beta coefficients for all four spacing conditions. The continuous line represents the best fit to the data (i.e., a logarithmic function). Error bars indicate 95% confidence intervals for each beta coefficient based on the linear mixed model used to compare spacing conditions.

To obtain an estimate of the time that a sequential memory trace can survive without being reinforced, we examined the evolution of the predictability score over the different repetition spacing. When the predictability score becomes null (i.e., when there is no longer any difference between unpredictable Position 1 and predictable Positions 2 and 3), then we can assume that the spacing between two repetitions is too important to consolidate the sequence's memory trace. We ran a regression analysis on the beta coefficients of each linear regression of the mixed model described above to determine which function best fit the dynamics we observed for the predictability scores (the greater the beta coefficient, the greater the learning of the sequence). The models were compared using AIC, BIC, and the *R* ^2^ scores (see Table 3). Lower AIC and BIC scores and higher *R* ^2^ values indicate a better model fit. Results revealed that the evolution of the predictability scores was better captured by a logarithmic function more than by a power, exponential, or linear function. Fig. 3 shows the results of the LME for the predictability score across the four spacing conditions, together with the logarithmic function for the distribution of the beta coefficients.

**Table 3:** Model comparison with parameter estimates for each model

Model	*a*	*b*	AIC	BIC	*R* ^2^
Logarithmic	0.015	0.001	−39.542	−41.383	0.937
Power	0.015	−0.071	−38.537	−40.378	0.901
Exponential	0.019	−0.088	−36.292	−38.133	0.889
Linear	0.019	−0.001	−35.487	−37.328	0.825

Abbreviations: AIC, Akaike's information criterion; BIC, Bayesian information criterion.

## 4 Discussion

In the present study, we used a combination of the SRT task (Nissen & Bullemer, 1987) and the Hebb repetition paradigm (Hebb, 1961), in which a variable number of random sequences were inserted between two repetitions of a target sequence to test the limit at which a sequential memory trace vanishes. To do this, we systematically manipulated the spacing between two repetitions of the target sequence by inserting zero to six random filler sequences. Consistent with previous studies (Malassis et al., 2018; Minier et al., 2016; Rey et al., 2022, 2019), we observed that baboons were able to learn sequences of three locations, as indicated by the decrease in RTs for the predictable second and third positions in the sequence across repetitions, even for the largest spacing. The observation of faster RTs for the third compared to the second position is also consistent with previous findings showing that the third position benefits from stronger contextual information during learning compared to the second (Elman, 1990). This advantage suggests that the occurrence of the third position is progressively predicted by the co‐occurrence of the first and second positions.

To our knowledge, the present study is the first to provide quantitative evidence on the role of repetition spacing in the formation of stable sequential memory traces by controlling for the influence of verbal consolidation strategies and by testing a nonhuman primate species (here, Guinea baboons, *P. papio*). More importantly, our results showed that learning of the sequence (measured by a predictability score) decreased as spacing increased, and that the relationship between spacing and sequence learning best fitted a logarithmic function. This result suggests that the memory trace of a sequence survives by following the nonlinear dynamics of a logarithmic function, and that even if we were to further increase the spacing between two repetitions of the target sequence, we should still observe learning of the sequence (manifested here by an increasing difference in RT between predictable and nonpredictable positions). This has important implications for current models of SL.

The idea that sequence learning is based on the creation and consolidation of chunks is supported by several influential chunking models (Frank et al., 2010; French et al., 2011; McCauley & Christiansen, 2019; Perruchet & Vinter, 1998; Robinet et al., 2011) and, to our knowledge, the results observed in the current study provide the first quantitative estimate of the lifetime of chunk representations. For one of the most influential models in this field, that is, the PARSER model, each time a new chunk is created in perceptual memory, this new unit receives a certain weight that materializes the strength of its trace in memory. This weight then decreases linearly if the chunk is not encountered and processed again. So, after a relatively short time (20 processing cycles in the original model), the memory trace of this chunk may disappear if it has not been processed again. However, our data suggest that each new sequence that is transiently memorized seems to survive much longer than predicted by the PARSER model. It would thus be important to see to what extent a much greater spacing of a repeated sequence (e.g., 20) would lead to learning of this sequence. Unfortunately, this type of experiment is too costly with the paradigm used here with baboons, because as Fig. 2 shows, we only start to observe a difference between predictable and nonpredictable positions from the third Block onward (i.e., after more than 300 repetitions of the target sequence) in the maximum spacing condition (i.e., 6F; requiring already a total of 2100 trials).

However, recent human data obtained in a Hebb lexical decision task (i.e., Pinto Arata, Ramisch, et al., 2024) indicate that learning is possible after a spacing of 60 (Pinto Arata, Ramisch, et al., 2024). Whereas in the present study, a logarithmic function provided the best fit to our results, Pinto Arata, Ramisch, et al. found that learning was best fitted by a power law. This is not surprising, since both functions have been shown to be highly correlated across different learning tasks (Radvansky, Parra, & Doolen, 2024). Indeed, if one fits well, the other is likely to do so as well. More crucially, this result validates the idea of a very slow disappearance of the sequence's memory trace, which appears to be captured by the dynamics of either a logarithmic or a power function.

Similarly, McCauley and Christiansen's (2019) recent model also assumes a mechanism for chunk creation as words and word sequences are processed, but unlike Parser, it assumes that these new chunks are never forgotten once they have been created. Even if there is no empirical data to validate this strong hypothesis, we can nevertheless consider that if the dynamics of forgetting the memory trace of a sequence follows a logarithmic function, then the trace decreases asymptotically and, as a result, its survival time is potentially very long. Our data, therefore, seem to be fairly consistent with the hypothesis formulated by this model concerning the extremely long survival of chunks that are created over time and exposure to language sequences.

The idea that these sequential memories have a very long lifetime can also certainly help to resolve the question of how infants are able to learn relatively infrequent words (Pinker, 1994; Swingley, 2008). Indeed, representations of words, even when being encountered relatively infrequently, can remain in long‐term memory for years once they are part of the mental lexicon. This would be linked to the undoubtedly general property of nonlinear decay of sequential memory traces, which follow a logarithmic function. The present study suggests that this general property is probably also present in other primate species, such as the Guinea baboon.

Beyond the long survival of sequential memory traces, it should be noted that in humans, with the development of motor control of the oral‐phonatory apparatus during the first year of life, young children are then able to generate verbal sequences themselves, producing repetitions that can compensate for the poverty of surrounding linguistic stimuli. By means of inner language, they can thus self‐refresh verbal sequential memory traces, increasing the strength of these memory traces and the efficiency of the processing of this information within their language perception and production systems (Chater, McCauley, & Christiansen, 2016).

While these findings contribute to our understanding of the temporal dynamics of memory trace formation, they call for further study, particularly because of our limited sample size. Specifically, 12 baboons completed all conditions and were, therefore, included in the final analysis. Nevertheless, each baboon performed 1000 trials per spacing condition on the repeated target sequence, resulting in a total of 100 trials per block, which gives us extremely robust averages per block and per monkey, as indicated by the very small confidence intervals. As such, even if future studies are needed both to replicate and extend our findings, the present dataset provides highly reliable estimates of the evolution of the strength of sequential memory traces as a function of the spacing between repetitions.

## 5 Conclusion

The present study demonstrates that a nonhuman primate species lacking language can maintain and consolidate a sequence of elements even when they are interleaved with a relatively large number of random sequences. These findings suggest that regularity extraction is possible in the absence of language and may rely on language‐independent associative learning mechanisms alongside language‐related refreshing mechanisms. Most importantly, the current study is the first to provide an estimate of the natural impact of forgetting on a sequential memory trace that does not benefit from verbal consolidation strategies. It indicates that the memory traces of sequential information certainly have a much longer lifetime than previously assumed.

## Conflict of interest statement

The authors declared no potential conflicts of interest with respect to the research, authorship, and/or publication of this article.

## Funding information

This research was supported by the Convergence Institute ILCB (ANR‐16‐CONV‐0002), the COMPO ANR project (#ANR‐23‐CE23‐0031), the HEBBIAN ANR project (#ANR‐23‐CE28‐0008), and the ERC advanced grant (#POP‐R 742141). For the purpose of Open Access, a CC‐BY 4.0 public copyright license has been applied by the authors to the present document and will be applied to all subsequent versions up to the Author Accepted Manuscript arising from this submission.

## Ethical approval and informed consent

This research adhered to the applicable French rules for ethical treatment of research animals and received ethical approval from the French Ministry of Education (approval APAFIS#2717‐2015111708173794 10 v3).

## Open practices statements

We report all data exclusions, all manipulations, and all measures and statistical tools in the study in the Method section. All data and analysis code are available online at https://osf.io/vdyt7/?view_only=893a3f4abee04513a98bb5939c4d1d66.

## Note

1 Inspection of the response times distribution revealed that a majority of responses were produced around 500 ms. A smaller group of RTs appeared around 1000 ms and was likely due to situations in which baboon's response was not recorded by the computer, because their hands were dirty. In this situation, they had to touch the screen again, and longer RTs were recorded (that are on average twice longer compared to the first responses). This is why we have adopted this two‐step trimming procedure.

## Appendix A

### A.1

**Table:**

	Second position in transition								
First position in transition	1	2	3	4	5	6	7	8	9
1		426	421	438	365	360	447	359	371
2	506		457	411	377	393	391	365	393
3	502	435		443	368	353	439	372	365
4	486	423	448		366	374	434	339	358
5	485	408	378	444		345	449	392	380
6	477	383	379	426	344		448	384	418
7	472	424	435	423	370	381		374	371
8	445	388	401	396	342	367	443		396
9	487	403	410	425	334	361	437	362	

## Appendix B

### The four selected repeated sequences and their respective mean TT1 and TT2 values

**Table:**

Triplet	TT1	TT2
**524**	408	411
**692**	418	403
**276**	391	381
**763**	381	379

Abbreviation: TT, transition time.
